# Heat Stress Perception among Native and Migrant Workers in Italian Industries—Case Studies from the Construction and Agricultural Sectors

**DOI:** 10.3390/ijerph16071090

**Published:** 2019-03-27

**Authors:** Alessandro Messeri, Marco Morabito, Michela Bonafede, Marcella Bugani, Miriam Levi, Alberto Baldasseroni, Alessandra Binazzi, Bernardo Gozzini, Simone Orlandini, Lars Nybo, Alessandro Marinaccio

**Affiliations:** 1 Centre of Bioclimatology (CIBIC), University of Florence (UNIFI), 50144 Florence, Italy; m.morabito@ibimet.cnr.it (M.M.); simone.orlandini@unifi.it (S.O.); 2 Department of Agriculture, Food, Environment and Forestry (DAGRI), University of Florence (UNIFI), 50144 Florence, Italy; 3 Institute of Biometeorology, National Research Council (IBIMET-CNR), 50145 Florence, Italy; 4 Occupational and Environmental Medicine, Epidemiology and Hygiene Department, Italian Workers’ Compensation Authority (INAIL), 00143 Rome, Italy; m.bonafede@inail.it (M.B.); m.bugani@inail.it (M.B.); a.binazzi@inail.it (A.B.); a.marinaccio@inail.it (A.M.); 5 Tuscany Regional Centre for Occupational Injuries and Diseases (CeRIMP), 50135 Florence, Italy; miriam.levi@uslcentro.toscana.it (M.L.); baldasse1955@gmail.com (A.B.); 6 Tuscany Region, LaMMA Consortium, Weather Forecaster and Researcher at Laboratory of Monitoring and Environmental Modelling for Sustainable Development, Sesto Fiorentino, 50019 Florence, Italy; gozzini@lamma.rete.toscana.it; 7 Department of Nutrition, Exercise and Sports, Section for Integrative Physiology, University of Copenhagen, 2100 Copenhagen, Denmark; nybo@nexs.ku.dk

**Keywords:** migrant, heat waves, heat perception, Wet Bulb Globe Temperature (WBGT), Universal Thermal Climate Index (UTCI), occupational risk

## Abstract

Climate change will increase the frequency and severity of hazard events such as heat waves, with important effects in several European regions. It is of importance to consider overall effects as well as specific impact on vulnerable population groups such as outdoor workers. The agricultural and construction sectors represent two strategic occupational fields that in relatively recent years involve an increasing number of migrant workers, and therefore require a better management of cultural aspects, that may interact with and impact on heat-related health risk. For this reason, the present study evaluated heat-stress perception and management among native and immigrant workers in Europe. As part of the EU’s Horizon 2020 HEAT-SHIELD project (grant agreement No. 668786), two agricultural and one construction companies, traditionally employing migrant workers, were evaluated with a questionnaire survey during the summer months of 2017. The data collected (104 case studies) were analyzed using descriptive statistics (Chi-squared tests) and the analysis of variance was performed with ANOVA test. From the results, migrant workers declared that work required greater effort than do native Italian workers (χ^2^ = 17.1, *p* = 0.001) but reported less impact from heat on productivity (χ^2^ = 10.6; *p* = 0.014) and thermal discomfort. In addition, migrant workers were mainly informed through written or oral communications, while native workers received information on heat-health issues through training courses. These findings are of importance for future information and mitigation actions to address socio-cultural gaps and reduce heat-stress vulnerability.

## 1. Introduction

Numerous studies have documented that the human-induced climate change has increased the frequency and severity of hazard events such as heat waves across the globe and recent studies evidenced that several areas of Europe are at high risk [1,2,3,4]. In particular, besides the Mediterranean region, several Western European regions and the Balkans could see increases of heat wave intensity in the 21st century [5,6,7]. The greater intensity and persistence of heat stress conditions to which the population will be subjected, therefore, urgently requires the implementation of efficient heat-related adaptation strategies, with particular attention to the most vulnerable population groups. Workers represent an important part of the population potentially at high-risk of heat exposure for many easily understandable reasons, with potential consequences for their health and work productivity [8,9]. Occupational exposures to high temperatures without sufficient protection may also increase the risk of heat-related illnesses and injuries [10], in particular for outdoor workers. Agriculture and construction sectors are the most exposed and are characterized by a high number of migrant workers with cultural aspects (religious, linguistic, adaptation) that contribute to further increase the risk [11].

Cultural aspects related to the ethnicity in workplaces represent certainly very important heat-related occupational vulnerability factors, even if, at the moment, they have not been investigated in depth. In particular, only a few studies have specifically addressed the issue of different cultural aspect related to the ethnicity, as a risk factor for heat-related human health [12] clearly indicating a knowledge gap which needs to be addressed in the face of climate change. An ethnic group is a category of people who identify with each other based on similarities such as common ancestry, history, country of origin, language, religious grounds, society or cultural tradition [13]. This aspect is of great importance given that, in many countries, specific occupational sectors prevalently involve migrant workers. In the past decade, in Italy, the presence of migrant workers increased by 80%: specifically, an increase from 1.4 million units in 2007 to 2.4 million was observed in 2016, when the number of Italian employees decreased by about one million units [14]. Moreover, the global economic crisis that also affected our country since the beginning of this century, further worsened the conditions of migrant workers, generally employed in precarious, laborious and risky, manual, low-tech and unskilled jobs, summarized as 3Ds jobs (dangerous, dirty and demanding/degrading work) that Italians are reluctant to perform [15]. In 2016, in Italy, positively assessed work injuries involved more than 61,000 migrant workers (15% of the total), of which more than 45,000 occurred to non-EU citizens (−14.4% compared to 2012) and about 16,000 to Community workers (−18.3%). The majority of the injured workers from the European Union come from Romania (61.3% in 2012–2016), while Moroccan (16.5%) and Albanian workers (13.4%) are the most affected non-EU citizens [16].

Despite growing attention by public opinion and companies on heat-related risks for workers’ health and safety, individual risk perceptions [17] constitute an important variable for illness and injuries prevention.

At European level, the ongoing HEAT-SHIELD project (https://www.heat-shield.eu/) has the mission to investigate the negative impacts of workplace heat-stress perception on health and productivity of workers employed in five strategic European sectors (tourism, agriculture, manufacturing, construction and transportation), with the aim to develop preventive solutions to protect the health and productivity in the work place from excessive heat. For this reason, in Italy, since summer 2017, some case studies have been organized, gathering information on topics related to the heat-stress perception and management collected through the submission of questionnaires to native and migrant workers employed in the agricultural and construction sectors. There is currently no information available on this topic, even if a significant increase in cultural diversity in the work population has been observed and, during periods of extreme heat, there may be disparities in the adaptive capacity of minority groups [18,19]. The main aim of this study is to investigate how cultural aspects can influence heat-stress perception and management among native and immigrant workers, in order to inform health care decision making aimed at reducing socio-cultural gaps and their influences on heat-stress vulnerability.

## 2. Materials and Methods

The study was carried out in Central Italy, in an area located to the south-west of the Apennine mountains and particularly, in the plane and low hill of the Provinces of Florence and Pistoia (Tuscany). This area is characterized by a sub-Mediterranean climate with hot and dry summer. As part of the HEAT-SHIELD project (European Union’s Horizon 2020 grant agreement No. 668786), the Italian partners selected some companies involved in the agricultural and construction sectors. The companies’ recruitment was carried out after a series of meeting with local stakeholders, including health authorities, trade unions, employers’ associations and associations of professionals responsible for control and vigilance within the work places.

Three companies of the agricultural and construction sectors, traditionally employing migrant workers, were identified, which also showed extreme interest in participating in the survey:

Palagio farm, operating in the wine and olive oil sectors since 2000, located in the municipality of Figline Valdarno (Florence Province). The estate has an extension of about 350 hectares and 18 farm workers involved in June and July are particularly busy in the pruning and lacing of the vines while from the middle of August and until the end of September they harvest grape. The daily working time is from 8:00 a.m. to 5:00 p.m., with 1-h lunch break, and no change in working hours is foreseen during the summer.

Oscar Tintori farm deals with the cultivation of citrus fruits in the greenhouse since 1970. The company is located in Pescia (Province of Pistoia) and it is divided into two units distant about 2 km from each other: the sale point and the area dedicated to crops. The organization of the company provides 12 workers employed in greenhouse activities and their daily working time during the summer is rescheduled (shifted by 2 h): from 6.00 a.m. to 2:00 p.m., with 1-h lunch break.

Temporary business associations set up for the construction of the tramway in Florence (Grandi Lavori Fincosit, Trafiter and Alstom). More than 300 construction workers were involved in the construction of the tramline on a large area of about 10 km in length and in one of the most urbanized areas of the city. During the summer period the daily working time is shifted by 1-h, starting work at 7:00 a.m. and finishing at 3:00 p.m.), with 1-h lunch break.

### 2.1. Workers Recruiment

The recruitment of workers to be involved in the study was carried out on a voluntary basis. All workers of the selected companies were given the opportunity to take part in the study, leaving free choice of adhesion to every single worker. The ethics committee of the University of Florence provided consent to conduct the questionnaire/data collection and analyze participants’ data. The ethics committee authorized the process of the worker’s personal data based on the Italian Legislative Decree 30.6.03 n. 196 of the Privacy Code. Each worker signed an informed consent in which the project aims and the workers ‘commitments required for the study were described.

### 2.2. Heat-Shield Questionnaire

A self-administered questionnaire survey (see Supplementary Material) was carried out in the summer months of 2017 in order to collect information on workers’ risk perception of heat stress in the workplace and possible productivity losses due to extreme heat. The survey (Annex 1) was an adapted version of the original one developed by Kjellstrom et al. within the “Hothaps programme”, a multi-centre health research and prevention programme aimed at quantifying the extent to which working people are affected by, or adapt to, heat exposure in the workplace, and climate change role in increasing such effects [20]. The original version was also used also by Dutta et al. to characterize the effects of heat on construction workers from a site in Gandhinagar, India [21]. The estimated time to complete the questionnaire was around ten minutes. The questionnaire is divided into 3 sections including the physiological characteristics of the subject, the information about the work activity performed and the workers’ heat perception.

In addition, safety measures to protect against extreme heat were assessed by asking workers to indicate whether any leaflet publications, information sessions or training sessions are available in the workplace, and their level of satisfaction regarding safety measures in place. The answers could vary on a four-point scale from “not at all satisfied” to “extremely satisfied”; in addition, the “unsure” answer option was also available.

For the purpose of the present study, only sections related to workers’ socio-cultural, educational and occupational context, to workers’ perception of heat stress and productivity losses due to extreme heat and to safety measures adopted in the workplace were taken into consideration in the statistical analysis.

### 2.3. Environmental Monitoring and Heat Stress Assessment

In each company, during the 2017 summer season, a microclimatic monitoring was carried out through the installation of a complete weathers station (HOBO U30 NRC) able to measure air temperature (°C), relative humidity (%), atmospheric pressure (hPa), black globe temperature (°C), wind speed (ms^−1^) and solar radiation (W/m^2^). In particular, the black globe temperature was measured inside a 150 mm diameter black globe (with emittance equal to 0.95) inside which a temperature sensor (pt100) is positioned and validated by the comparison with a standard WBGT heat stress monitor instrument. The shape, the size and emissivity of this globe are chosen so as to simulate the human body and the relative convective and radiative exchanges with the surrounding surfaces. In outdoor environments, radiative exchanges depend on solar radiation (direct and diffuse) and on the heat flow emitted by radiation from surfaces at a given temperature. The solar radiation was measured by silicon pyranometer sensor that offers a measurement range of 0 to 1280 W/m^2^ over a spectral range of 300 to 1100 nm. Wind speed was measured by a “Wind Speed Smart Sensor” that provides data reporting average wind speed (from 0 to 76 m/s) and highest 3 s gust for each logging interval. Air temperature and relative humidity was measured by a 12-bit Smart Sensor (temperature range −40 °C to 75 °C).

These data were used to evaluate thermal stress conditions in the workplaces. Two biometeorological indicators, the Universal Thermal Climate Index (UTCI) [22] and the Wet Bulb Globe Temperature (WBGT) [23,24] index was assessed. In particular, WBGT was calculated using the heat stress calculation tool provided by the Climate Chip (Climate Change Health Impact & Prevention) web-platform (http://www.climatechip.org/), instead the UTCI was calculated by using the UTCI software code “version a 0.002”, freely available online (http://www.utci.org/). Both indices were calculated using the microclimatic parameters measured by the weather station.

The UTCI represents the state-of-the-art of thermal-stress assessment, while the WBGT is a thermal stress indicator specifically used for the working environment and that allow to provide useful suggestions on the work-rest scheduling. In particular, the WBGT index represents a reference standard used by international organizations involved in the protection of workers’ health [24,25,26], and also for this reason this index was selected as a reference in the European project HEAT-SHIELD.

It is however important to consider that both indices are expressed in °C but, because different methodologies were adopted to develop these biometeorological indicators, different heat-stress scales represent the results of these indexes, higher for UTCI than WBGT.

### 2.4. Statistical Analysis

This study analyzed data of 104 case studies conducted during summer 2017 (from May to September). Within 3 companies in Central Italy, a monitoring on critical and non-critical summer days, that covered environmental, behavioral and perception parameters, was carried out.

The data collected were analyzed using descriptive statistics (frequency, mean, standard deviation) and analytical tests. Chi-squared tests were used to determine the association between the nationality and some variables related to the perception of heat and effort. The statistical significance of differences in mean scores by nationality was calculated using ANOVA test. Missing data were used only in descriptive analysis, not in statistical tests. All analyses were performed by using SPSS version 22.0 [27]. The statistical significance was set at *p* < 0.05.

## 3. Results

### 3.1. Microclimate and Heat Stress

**Figure 1:**
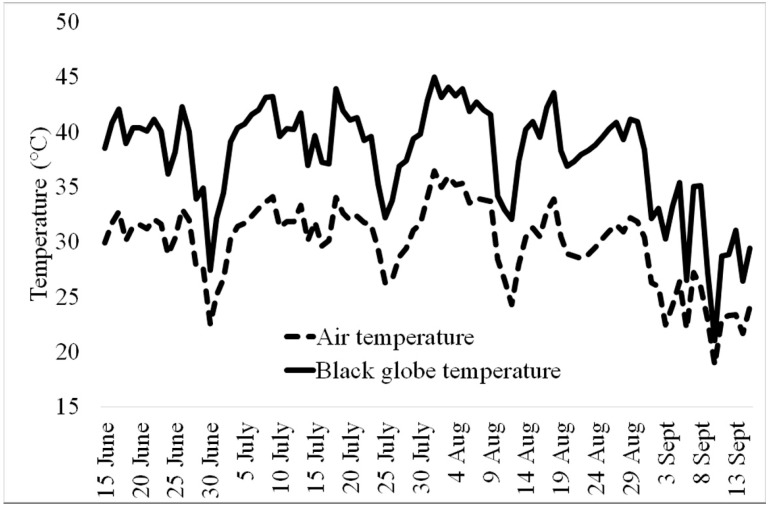
Air temperature (continuous line) and black globe temperature (dashed line) measured during the working time of the day (8:00 a.m.–5:00 p.m.) in the three work sites involved in the study during the summer 2017.

The environmental monitoring has shown average values of air temperature during the typical working time (from 8.00 a.m. to 5.00 p.m.), ranging between 14.5 °C and 36.5 °C (dashed line in Figure 1).

**Figure 2:**
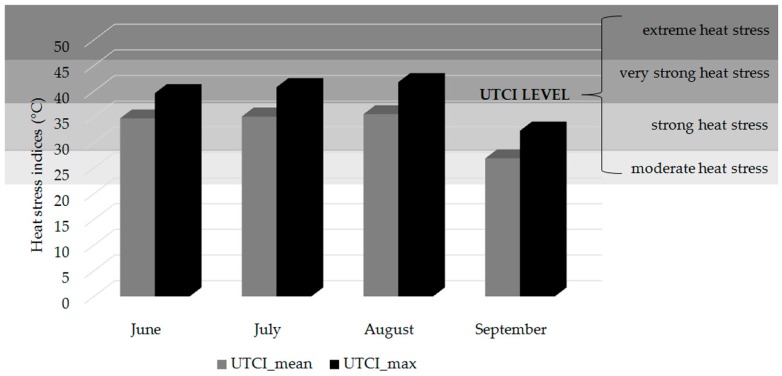
Mean and Maximum Universal Thermal Climate Index (UTCI) index for each month during the working time at the three work sites involved in the study (summer 2017) and the recommended rest according to the WBGT ISO standard for a worker that perform an activity that requires an effort of 300 watt. The bands of different shades of gray indicate instead the heat stress thresholds according to the Universal Thermal Climate Index (UTCI).

During the studied period, well-defined periods with a persistent daily average air temperature above 32 °C were clearly identified, corresponding to four heat waves that affected a large part of southern Europe, including the Tuscany region, during the summer season of 2017. Black globe-temperature values (continuous line in Figure 1), which take into account the radiative contribution, were always higher than air temperatures, with peaks near 45 °C in the first ten days of August. In Figure 2, the average and maximum monthly values of WBGT during the working time were shown together with the recommended rest times in the hour according to the WBGT ISO standard [25,26].

The highest thermal stress UTCI values were recorded in August (41.8 °C), while the lowest values in September (32.3 °C). Considering a worker who performs an activity that requires an average effort of 300 watts, the ISO standard WBGT would have required an average break of 30 min in August, instead no breaks during working hours would be necessary in September. As for the months of June and July, the maximum UTCI during working hours was close to 40°C (39.6 °C and 40.8 °C respectively) and would have required an average break of 15 min per hour. If, on the other hand, daily mean values are taken into account, the heat stress value calculated according to the WBGT ISO standard would not require rests despite the equivalent temperature identified according to the UTCI index identifies a heat stress level. This is because the average value causes information about the worst conditions that occurred during the day to be lost. In practice, the highest WBGT values that occur during the central hours of the day are averaged with WBGT values recorded in the early morning hours, thus providing an average value that tends to underestimate the conditions that actually occur in the warmest hours.

**Figure 3:**
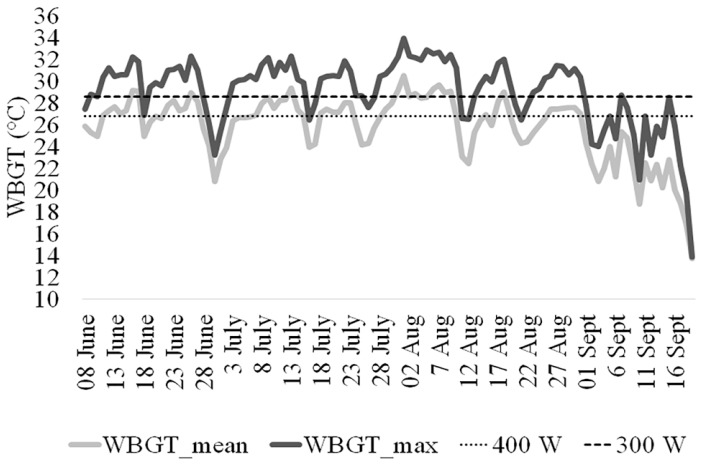
Mean and maximum daily Wet-Bulb Globe Temperature (WBGT) index during the working time of the day at the three work sites involved in the study, summer 2017. The dashed lines represent the WBGT ISO standard thresholds respectively for a high (400 W) and a moderate (300 W) work effort as declared by the native workers.

Figure 3 shows WBGT values (maximum and mean) and the risk thresholds that required a behavioral modification to counteract the heat stress according to the American Conference of Governmental Industrial Hygienists (ACGIH) for acclimatized workers engaged in moderate (300 W) and high (400 W) work efforts. It is clearly evident that most of the average thermal conditions monitored during the studied period required behavioral actions for a worker involved in high work efforts, while for moderate activities actions were generally required if workers were exposed to the maximum thermal stress conditions (Figure 3).

### 3.2. Differences between Native and Migrant Workers

The total number of workers in the selected companies was 330 and among them, those who agreed to participate in the study, were 104 (96 men and 8 women) from 3 Tuscan companies: two in agriculture sector (outdoor, *n* = 16; greenhouse, *n* = 10), and one in construction sector (outdoor, *n* = 78). Table 1 shows the distribution of workers by place of birth and sector.

Among migrants, the largest group (*n* = 22) consists of the Albanian workers employed in the construction sector.

The average age of participants was 46.7 years (*SD* = 9.6) for native, and 41.8 (*SD* = 6.5) for migrant workers (Table 2).

In terms of age, the largest age group was of that of workers over 50 years for natives (*n* = 28, 42.4%) and the one between 40 and 49 years old for migrant workers (*n* = 20, 52.6%). There were 18 natives and 14 migrants aged less than 39. Most of both natives (*n* = 50; 87.7%) and migrants (*n* = 29; 80.6%) had a middle or high school diploma. Most claimed that their income was in line with the one of the companies in same sector (19 natives, 28.8%, 9 migrants, 23.7%) and were not seasonal workers (50 natives and 36 migrants).

As shown in Table 3, which compares the scores assigned to different items by nationality based on the Chi-square test, compared to native workers, migrant workers reported a higher physical effort (χ^2^ = 17.1, *p* = 0.001).

In particular, most of them declared a high effort while, on the contrary, natives declared a moderate effort. On the basis of perceived and declared physical exertion, migrant workers reached the heat risk threshold (WBGT ≥ 27.9 °C) more easily than native workers (WBGT ≥ 29.3 °C) in the period May–September 2018. This result is observed in terms of both maximum and average WBGT values (Figure 3).

The heat perceived during work in the presence of a heat wave was however greater for native workers (χ^2^ = 13.9; *p* = 0.008), as well as the perception of the decline in productivity (χ^2^ = 10.6; *p* = 0.014). Most of workers (60%) that did not experience a loss of productivity were migrant. Native workers also reported to become more informed about the behaviors to be adopted during heat waves through safety courses (65% of natives) compared to migrant workers (χ^2^ = 21.15; *p* = <0.001). This latter, instead, declared to have been more informed through written (18.4%) or oral news (34.2%). Only 5.3% answered that they were not informed, 1 native and 4 migrants. However, migrant workers claim to be more satisfied than Italian workers with measures currently adopted in their workplace for reducing the effects of heat (χ^2^ = 39.58; *p* = <0.001). There is no statistically significant association between nationality in receiving advises when heat waves are in progress (χ^2^ = 0; *p* = 0.994).

The results of ANOVA test (Table 4) showed a significant difference between native and migrant workers in terms of the number of years they have been working in that sector (*p* < 0.001).

In addition, a significant difference was observed between natives and migrants regarding the number of hours worked outdoors in the summertime (*p* = 0.01). The number of hours (on average) worked indoor in the summertime is also different (*p* < 0.01).

## 4. Discussion

This study represents one of the first to assess how heat-stress perception in work place is influenced by socio-cultural aspects. Knowledge of the working conditions and occupational health of immigrant and ethnic minorities is important for initiating preventive and integrational efforts. The interviewed migrants in this study declared to carry out works that require greater effort than do native workers, it’s consistent consistently with the representation of immigrants in low-skilled, high-risk manual jobs [28]. Immigrants tend to be healthier upon arrival than natives, although this health advantage declines over time [29], therefore might hold more physically strenuous jobs than natives. These physically strenuous jobs are prevalent in sectors like construction, meatpacking, and agriculture [30]. Indeed, migrant workers are also on average younger and with less work experience in the specific sector, and in addition, during summertime, they usually work outdoors more hours per day [31]. Furthermore, the different perception of job risk, linguistic barriers and cultural factors that reduce the effectiveness of any training, make migrant workers probably less able to negotiate the type of tasks they perform than native workers [32]. However, migrants claim to perceive less heat and to experience a lower productivity drop compared to native workers. This is probably because migrants have a higher heat tolerance threshold or a poorer perception of health risk, although the social desirability bias cannot be excluded: the greater job insecurity experienced by migrant workers might have influenced the answers provided [18,32].

An important dimension of job quality is related to occupational health and safety system in place. A relevant result of this study is related to the information and training provided by employer or adviser during heat waves on how to carry out work activities. Migrant workers claim to mainly be informed through written or oral communications, while native workers mainly through training courses. As for migrant workers, the difficulty in understanding the language is an important factor in the perception of the heat risk in the workplace, our results suggest the need to implement measures specifically targeting migrants. In particular, health and safety training, taking into account language difficulties, cultural and religious aspects, should be promoted in sectors where migrants are more widely employed [12,31]. Particular attention should also be paid to encourage the use of personal protective equipment and, if possible, realized with materials that do not increase the heat perception. Moreover, the results show that migrants are more satisfied than native workers with measures adopted in their workplace for reducing the heat effects. The greatest satisfaction could be explained by previous experiences made by migrant workers in their countries of origin with health and safety systems worse than the native one. Special measures to increase awareness of safety rights in the workplace, especially in sectors with a high level of injury and lower perception of risk, are also required [31].

The main strength of this study is that it is the first attempt to investigate heat related perception from the perspective of workers through self-completion questionnaires. It is important to understand workers’ perceptions of extreme heat exposure in workplace, as this information may provide evidence for updating heat prevention strategies to reduce the impact of climate change on workers’ health and safety. The prevention strategies also include the creation of specific behavioral guidelines for the working sector, calibrated for the different occupational sectors. Within these, particular importance should be given to maintaining a good level of hydration of the subject, not only during the performance of the work activities but also outside of working hours, taking up many liquids and foods with high water content and rich in mineral salts such as fruits and vegetables, [33], as well as avoiding alcoholic beverages that further exacerbate dehydration. Recent studies show that, during the summer, the level of dehydration is already very high even before starting work. In particular, some monitoring carried out on workers (urine sampling) showed that most of them were already strongly dehydrated before starting their day’s work [34,35]. This entails a strong stress and also causes an alteration of the perception of effort and therefore of risk [36]. It is evident that the dietary habits that underlie the maintenance of a good level of hydration and nutrition are strictly dependent on cultural aspects (e.g., subjects of Muslim origin are at greater risk during the Ramadan period) [37]. The results of a recent study showed that from the Eastern-Mediterranean Region workers exhibit a significantly increased risk for occupational injuries during Ramadan in periods characterized by heat-waves, while their frequency was somehow reduced for days associated with Ramadan characterized by increased but not extreme temperatures [38].

The main limitation of this study is the limited and unbalanced sample (just over 100 workers of which 63% are natives). Moreover, the migrant group is not homogeneous, being prevalently composed by Albanians that work in the constructions sector, whereas the 25% of the sample that works in agriculture is represented by North Africans. Nevertheless, the study managed to highlight statistically significant differences, supporting the fact that cultural diversity issues in the workplace should be seriously taken into consideration in the coming years. In order to avoid bias in the results, we should not consider immigrants as a homogeneous group of individuals and the specificity of each nationality should be taken into account. Therefore, with a different sample, further information could be obtained. In addition, we must also consider that migrant workers are younger than Italian, and this could imply a different perception of heat and efforts. It is well known that the main reason for immigration is economic opportunity, and that migrants are generally younger and an important fraction of the active population in Italy. Furthermore, they are often less qualified job seekers, and may be particularly at risk as they are often less qualified than their native counterparts and could be subject to employment discrimination [39]. Another potential bias is the underreporting due to communication difficulties during the interview and to social desirability bias, particularly frequent among migrant workers concerned about possible reprisal or staying away from work too much time [40].

## 5. Conclusions

In the future the increasingly effects of climate change will make necessary mitigation strategies to face the effects of high temperatures on the population, especially the most vulnerable categories, including workers.

Our findings are important for promoting and regulating prevention measures related to heat waves and their impact on workers. In addition, climate change is expected to trigger growing population movements within and across borders, as a result of such factors as increasing frequency and intensity of extreme weather events and, for this reason, the number of migrant workers will tend to increase further in the coming years. Because of cultural differences compared to their places of origin, these workers may perceive the risk related to high temperatures in the workplace differently than native workers.

This study shows that there are ethnical differences concerning the perception of effort and heat, as well as about information on how to deal with it. The low proportion of respondents unsatisfied with current measures adopted to inform on and reduce the effects of heat, recommends a better attention of employers to their workers’ health and safety.

For informing on and reducing the effects of heat, indicates a good attention by employers on the health and safety of their workers. However, it is necessary to take into consideration that the migrant workers have greater job insecurity, compared to native ones, and so the possible fear in answering to the questionnaire should not be underestimated.

For the future, it will be necessary to create larger and more homogeneous samples to make ethnic comparisons also effective regardless of the age, type of job and country of origin. However, these preliminary results already highlight the strong need to intensify training courses for migrants, which should take into consideration linguistic barriers as well as cultural and religious differences. Religious aspects, in fact, have not yet been considered but they could be an important variable that regulates the habits in drinking and eating, thus influencing the state of health of workers.

## Figures and Tables

**Table 1:** Workers by birth place and sector.

Workers		Agriculture	Construction	Total
Birth place	Italy	17	49	66
	Albania	2	22	24
	Romania	4	3	7
	Moldova	1	2	3
	Morocco	1	1	2
	Germany	1	1	2
	Total	26	78	104

**Table 2:** Sample characteristics and statistical associations.

Workers		Native Workers		Migrant Workers			
		*n* ^a^	% ^b^	*n* ^a^	% ^b^	χ^2^ ^c^	*p* ^d^
**Gender**	Male	60	90.9	36	94.7	0.498	0.481
	Female	6	9.1	2	5.3		
Age groups	≤39	18	27.3	14	36.8	11.818	0.003
	40–49	20	30.3	20	52.6		
	≥50	28	42.4	4	10.5		
Level of education	Apprenticeship	1	1.5	2	5.3	6.04	0.11
	Trade school	2	3.0	5	13.2		
	Secondary	37	56.1	17	44.7		
	Higher secondary	13	19.7	12	31.6		
	Missing	13	19.7	2	5.3		
Income	Below the average of the work country	2	3.0	0	0.0	3.053	0.217
	Within the average	19	28.8	9	23.7		
	Above the average	0	0.0	1	2.6		
	Missing	45	68.2	28	73.7		
Seasonal worker	Yes	7	10.6	1	2.6	2.643	0.104
	No	50	75.8	36	94.7		
	Missing	9	13.6	1	2.6		
Type of industry work environment	Agriculture outdoor	9	13.6	7	18.4	9.233	0.01
	Agriculture greenhouse	8	12.2	2	5.3		
	Construction	49	74.2	29	76.3		

^a^ Number of workers for each category; ^b^ Percentage of workers for each category; ^c^ Chi-squared test value; ^d^ *p* value significance.

**Table 3:** Chi-squared analysis results of the first part of the questionnaire submitted to workers.

Question	Answer Options	Native Workers		Migrant Workers			
		Mean	*SD* ^a^	Mean	*SD* ^a^	χ^2^ ^b^	*p*
How physically demanding is your job?	Light (1)–Moderate (2)–Heavy (3)–Very heavy (4)	2.58	0.767	2.93	0.815	17.129	0.001
How do you perceive the temperature while working during heat waves?	Neither warm nor cool (1)–Slightly warm (2)–Warm (3)–Hot (4)–Very hot (5)	4.31	0.731	4.06	0.719	13.924	0.008
Do you notice that you are less productive during a heat wave (e.g., you need more energy for the same work)?	No (1)–Yes, for less than 10% (2)–Yes for 10% to 30% (3)–Yes, for more than 30% (4)	2.43	0.708	2.17	0.814	10.57	0.014
Have you ever been informed by your employer or adviser how to act during heat waves?	No (1)–Yes, through written and oral news (2) –Yes, through safety courses (3)	2.64	0.496	2.32	0.658	21.15	<0.001
Do you receive warnings and advice from your employer or adviser during heat waves?	No (1)–Yes (2)	1.75	0.4	1.67	0.5	0	0.994
Are you satisfied or dissatisfied with measures currently adopted in your workplace for reducing the effects of heat?	Dissatisfied (1)–Undecided (2)–Satisfied (3)–Strongly satisfied (4)	3.4	0.827	3.5	0.641	39.581	<0.001

^a^ Standard deviation; ^b^ chi-squared test value; ^c^ *p* value significance.

**Table 4:** ANOVA analysis results.

Question	Native Workers		Migrant Workers			
	Mean	*SD* ^a^	Mean	*SD* ^a^	*F*	*p* ^b^
How many years have you been working in this sector?	19.24	9.427	12.62	5.445	44.737	<0.001
How many hours per day do you usually (on average) work outside in the summertime?	5.23	3.835	6.31	3.246	6.732	0.01
How many hours per day do you usually (on average) work outdoor in the summertime?	2.9	3.789	1.74	3.281	6.861	0.009

^a^ Standard deviation; ^b^ *p* value.

## Supplementary Materials

The following are available online at https://www.mdpi.com/1660-4601/16/7/1090/s1, General anonymous questionnaire: workers’ risk perception of heat stress in the workplace.
