# Supplementary material for: Heat Stress Perception among Native and Migrant Workers in Italian Industries—Case Studies from the Construction and Agricultural Sectors
Source: Int J Environ Res Public Health. 2019 Mar 27;16(7):1090. doi: 10.3390/ijerph16071090 (PMC6479923; doi:10.3390/ijerph16071090)
Supplement: Supplementary file 1 [file ijerph-16-01090-s001.pdf]

This project has received funding from the European Union's Horizon 2020, Grant Agreement No 668786

General anonymous questionnaire:- workers' risk perception of heat stress in the workplace

ID ……………………………….

Gender

☐ Male ☐ Female

Date oh birth: ……./……./…….

Birth place: …………………………….

Country where you work……………………..

Height (cm)……………………………………**Weight (Kg)**…………………………….

Educational level

☐ Apprenticeship
☐ Professional school
☐ Secondary school
☐ High school
☐ Bachelor's/master's degree
☐ Phd

☐ Other ………………………………………………

Is your income below, above or in the average of the country where you are working?

☐ Below average ☐ Average ☐ Above average

What effort does your work require?

☐ Low (predominantly sitting and engaged in office work)

☐ Moderate (work supported with hands and arms: driving tractors or other machines, hammering, filing)

☐ High (intense work with arms and trunk: digging with shovel, sawing, planing, shearing grass by hand)

☐ Very high (very intense activity; manual handling of heavy loads)

Are you a seasonal worker?

☐ Yes ☐ No

In which sector do you work?

☐ Agricultural

☐ Construction

☐ Trasportation

☐ Manufacturing

☐ Turism

☐ Other……………………………………

How many years have you been working in this sector?

…………………………………………………….

How many hours a day do you work outdoors during the summer?

…………………………………………………….

How many hours a day do you work indoors during the summer?

……………………………………………………..

When you work in an indoor environment, is there a conditioning system?

☐ Yes ☐ No ☐ I don't know

Do you usually have work shift?

☐ Yes ☐ No

Do you suffer from chronic illnesses (a multiple answer is possible)?

☐ No

☐ Diabetes

☐ Hypertension

☐ Diseases of the respiratory system

☐ Allergies that are accentuated during particularly hot periods

☐ Kidney diseases

☐ Other ……………………………………….

During which work activities do you feel the greater impact of heat wave?

……………………………………………………………………………………….

What thermal sensation do you feel while working during a heat wave?

☐ Neither hot nor cold

☐ Slightly hot

☐ Moderately hot

☐ Hot

☐ Very hot

What impact does a heat wave have on your work (a multiple answer possible)?

☐ No impact

☐ Impact on well-being

☐ Impact on concentration

☐ Impact on productivity

☐ Health impact

☐ Other……………………………….

Do you experience a drop in productivity during a heat wave?

☐ No

☐ Yes, about 10%

☐ Yes, from 10 to 30%

☐ Yes, more than 30%

Have you ever been informed, by your employer or your manager, on how to behave in your work activities in the event of a heat wave (a multiple answer is possible)?

☐ No

☐ Yes, leaflet

☐ Yes, orally

☐ Si, posters

☐ Si, safety courses

☐ Other……………………………………………..

Receive warnings from your employer or manager about the possibility of a heat wave occurring (a multiple answer is possible)?

☐ No

☐ Yes, leaflet

☐ Yes, orally

☐ Yes, posters

☐ Other………………………………………………….

Are you satisfied / dissatisfied with the measures taken at the workplace to reduce the effects of heat?

☐ Very dissatisfied

☐ Dissatisfied

☐ I don't know

☐ Satisfied

☐ Very satisfied

What symptoms do you usually experience during the summer (a multiple answer is possible)?

☐ None

☐ Excessive sweating

☐ Tiredness / weakness

☐ Thirst

☐ Dizziness

☐ Disorder

☐ Stress increasing

☐ Discomfort

☐ Other……………………………………………

Have you ever been affected by the following diseases or symptoms during a heat wave (a multiple answer is possible)?

☐ Headache

☐ Weakness

☐ Rash

☐ Muscle cramps

☐ Fainting (heat syncope)

☐ Nausea or vomiting

☐ Heat cramps

☐ Heart attack

☐ None of the previous ones

☐ Other………………………………………………………….

Have you ever been hospitalized for one of the problems listed above?

☐ Yes ☐ No

If you answered YES to the previous question, specify which one.

………………………………………………………………………………

Does your work require a special clothing (for example, for reasons of protection or safety)?

☐ No

☐ Yes but does not accentuate the heat discomfort during a heat wave

☐ Yes, It is determined an accentuation of the heat discomfort during a heatwave

How do you try to reduce exposure to heat (a multiple answer is possible)?

- ☐ The exposure can not be reduced
- ☐ Increasing the number of breaks
- ☐ Using a cooler place for breaks (eg place in shade)
- ☐ Wearing more suitable clothes (light and breathable clothing)
- ☐ Drinking more water
- ☐ Moving work activities early in the morning or in the evening
- ☐ Other…………………………………………………………….
